# Effects of Lane Width, Lane Position and Edge Shoulder Width on Driving Behavior in Underground Urban Expressways: A Driving Simulator Study

**DOI:** 10.3390/ijerph13101010

**Published:** 2016-10-14

**Authors:** Shuo Liu, Junhua Wang, Ting Fu

**Affiliations:** 1 School of Transportation Engineering, Tongji University, 4800 Cao’an Highway, Shanghai 201804, China; 15521@tongji.edu.cn; 2 Department of Civil Engineering and Applied Mechanics, McGill University, Montreal, QC H3A 0C3, Canada; ting.fu@mail.mcgill.ca

**Keywords:** underground urban expressway, lane width, lane position, shoulder width, driving behavior

## Abstract

This study tested the effects of lane width, lane position and edge shoulder width on driving behavior for a three-lane underground urban expressway. A driving simulator was used with 24 volunteer test subjects. Five lane widths (2.85, 3.00, 3.25, 3.50, and 3.75 m) and three shoulder widths (0.50, 0.75, and 1.00 m) were studied. Driving speed, lane deviation and subjective perception of driving behavior were collected as performance measures. The results show that lane and shoulder width have significant effects on driving speed. Average driving speed increases from 60.01 km/h in the narrowest lane to 88.05 km/h in the widest lane. While both narrower lanes and shoulders result in reduced speed and lateral lane deviation, the effect of lane width is greater than that of shoulder width. When the lane and shoulder are narrow, drivers in the left or right lane tend to shy away from the tunnel wall, even encroaching into the neighboring middle lane. As the lane or shoulder gets wider, drivers tend to stay in the middle of the lane. An interesting finding is that although few participants acknowledged that lane position had any great bearing on their driving behaviors, the observed driving speed is statistically higher in the left lane than in the other two lanes when the lane width is narrow (in 2.85, 3 and 3.25 m lanes). These findings provided support for amending the current design specifications of urban underground roads, such as the relationship between design speed and lane width, speed limit, and combination form of lanes.

## 1. Introduction

With the fast development of urban traffic and the limitation of land use, the trend of building underground urban expressways has increased over the past few years. Many cities have built underground urban expressways, for example, Willits City Bypass in California Route101, Kallang-Paya Lebar Expressway (KPE) in Singapore, and the Bund Bypass in Shanghai. Several studies have shown the benefits of the underground road, such as protecting the environment from traffic noise and pollution, saving land resources for other purposes, and reducing traffic at transport nodes and in central business districts [1,2].

Driving along a dark, narrow environment can cause drivers anxiety and uncertainty, and thereby result in accidents [3,4]. In this situation, most drivers reduce their speed and increase their lateral distance to the tunnel wall [5]. The risk of accidents in a tunnel is approximately half of that on an open road; however, tunnel accident severity is higher for several reasons, e.g., tunnels make it easier for drivers to get seriously injured by crashing against the tunnel wall compared to the safety barrier in open roads. Tunnels also have a limited access to rescue devices such as cranes [6].

Road alignment, especially its cross-section factors such as lane width, lane position and shoulder width, has a significant impact on driving behavior. Many studies have been carried out on investigating the effects of lane width, lane position and shoulder width on driving behavior.

Researchers have shown that vehicle speed increases with the increase in lane and road width [7,8,9,10], and at the same time, the lateral position of the vehicle moves towards the outside of the lane [11]. Horst and Hogema found that drivers on the outside lane drive more towards the inside of the road on a wide road as more space to the wall is available [12].

Meanwhile, higher speed and larger lane deviation would lead to higher risks. Wilde proposed that drivers had an internal target level of risk and would change their action to reduce the difference between the perceived level and their target level [13]. Wider lanes were considered to decrease perceived risk, so drivers would take risky behaviors, such as acceleration and large lane deviation. Because of the monotonous environment, speeding often occurs in underground urban expressways leading to many accidents [14]. Several studies indicated that narrowing lanes is a measure to slow down the speed because a narrow lane may increase perceived risk [15,16].

Roadway shoulders have several functions, such as emergency stop and recovery areas for driver errors. Shoulder width also has effect on driving performance. On the one hand, narrow shoulders can create a dangerous situation by increasing the likelihood of off-road collision, and also increasing driver workload [17]. On the other hand, several studies have found that narrow shoulders would lead drivers to be more cautious and slow down to avoid danger [18,19]. Because of the limit of cross-sectional width, shoulders in underground urban expressways are not wide in general.

Lane position also affects driving behavior. Studies have shown that the difference between the tunnel wall located to the left and to the right is statistically significant on driving behavior. Drivers positioned the car further from the side line when the nearest tunnel wall was on their left side. The speed was lower on the right lane than on the other two lanes [19]. On the contrary, some scholars found that vehicles on the right lane kept a larger distance to the wall than those on the left lane [20,21].

Most of these studies have looked into open roads. Few studies have examined the effect of road alignment on driving behavior on underground urban roads, where drivers may act differently due to the high mental load of driving under low visibility and spatial pressure [22].

Most of the previous studies considered the three independent measures separately. A counterbalanced experimental design was used in this study, in order to consider all other possible factors. It included 15 possible combinations of the three independent measures.

Compared to research on cross-section of regular roads, cross-section factors of underground urban expressways have been less studied. Underground urban expressways include varied components, such as lane and shoulder width, horizontal and vertical alignment. All the factors can affect driving behavior [23,24]. Therefore, the method of studying driving performance must combine various conditions and is difficult to test on a full scale. The driving simulator can be effectively utilized to accomplish these tasks. In other words, one can control the change in road conditions easily and obtain data from many subjects [25]. The validity of some advanced driving simulators for the analysis of driving behavior in urban tunnels has already been confirmed in several studies [26,27]. Some researchers found that mean speed in a simulator was not exactly reproduced in a field study; however, the direction and magnitude of the speed in simulation experiments was similar with those in field studies [28]. In our previous research, the relative validity of Tongji simulator on studying speed and lane deviation of underground expressway was confirmed [29]. It was also proven by a simulator experiment that inside shoulder width has statistically significant effects on a vehicle’s lane position, and a vehicle’s lane position is negatively correlated to inside shoulder width [30].

With help of an eight-degree-of-freedom simulator, this paper investigates the effects of the three roadway design features on driving behavior, including speed and lane deviation, to provide reference and evidence for urban underground expressway alignment design specifications.

## 2. Methods

### 2.1. Participants

The study included 24 subjects: 12 male and 12 female. All the subjects were licensed drivers with normal or corrected-to-normal vision and between the ages of 23 and 51 (mean = 34 years, Standard Deviation (SD) = 8.6). Each participant had over three years of driving experience. Eighteen of the drivers had used the same driving simulator before; the other six had no driving simulator experience. To ensure the reliability of the experiment and avoid negativity from the participants, participants were asked to sign a consent declaiming that they will take the tests seriously and act as they do in real traffic conditions as much as possible.

### 2.2. Apparatus

**Figure 1:**
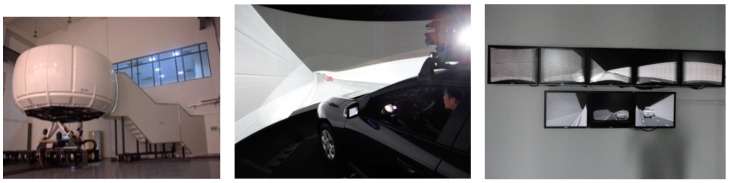
The driving simulator.

The driving simulator of Tongji University, as shown in Figure 1, is a motion-base simulator with eight degrees of freedom (DOF). A real car is placed in the middle of the experimental cabin as the test vehicle. A visual system, with five projectors, projects the road model on the spherical curtain. The simulator provides a 250° (horizontal) × 40° (vertical) forward view of the roadway from the driver’s position. The performances of the motion system, feedback systems and the cockpit have reached the international advanced level. The simulator is monitored by the controlling software (Scaner™ Studio v1.4 by OKTAL, Paris, France) for system control, scene creation, data acquisition, data analysis, etc. The software enables the creation of accurate models and a variety of scenarios. The validation of the Tongji simulator in researching underground road driving behavior has been verified in several published studies [14,29].

The geometry data of the bypass expressway in the study were from the actual design of a one-way three-lane underground urban expressway (the Bund Bypass, Shanghai, China), the length of which is about 3.6 km. It contained five horizontal curves, and the minimum curvature radius was 500 m. The minimum radius of vertical curve was 1200 m. The maximum longitudinal slope was 5%. The vehicle model was a regular four-door sedan, with a width of 1.8 m and a length of 5 m. To avoid interference with the driving behavior of the participant, no other vehicles were placed in the lane in which the test car was driving in all the scenarios [31]. A free traffic flow of 550 pcu/h in other lanes were set in the scenarios to create a realistic environment.

### 2.3. Variables Studied

**Figure 2:**
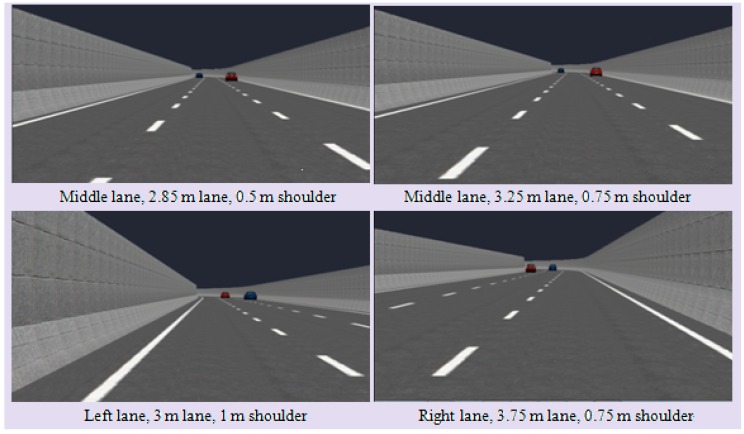
Examples of simulation scenario.

The impact on the driving behavior of the participants was studied in terms of three independent road alignment variables including lane width, shoulder width, and lane position. According to urban expressway design specifications in China [32,33], five lane width values are suggested—2.85, 3.0, 3.25, 3.5 and 3.75 m—and three shoulder width values are suggested—0.5, 0.75 and 1.00 m. Scenarios with different combinations of these suggested lane width and shoulder width values were tested. Figure 2 shows snapshot examples of the simulation scenario.

### 2.4. Data Extraction

#### 2.4.1. Objective Data

**Figure 3:**
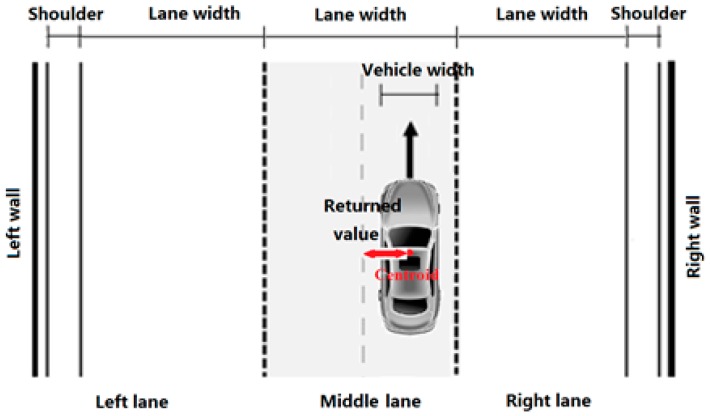
Definition of the lane deviation.

In the experiment, driving speed (km/h) and lane deviation (m) of the vehicle were recorded by the driving simulator with a sampling frequency of 10 Hz. In this study, lane deviation is defined as the offset between the position of the vehicle centroid and the centerline of the lane, as presented in Figure 3—a value of zero means that the position of the vehicle centroid aligns with the centerline of the lane. The lane deviation value is positive when the vehicle centroid falls on the left side of the centerline of the lane, otherwise it is negative.

Lane deviation was used to evaluate safety. Based on the positions of the vehicle and lane-marking, lane deviation was classified into three types, including: (1) regular situation, in which the vehicle stayed strictly inside the lane marking (in such a situation, the interference of the vehicle on other vehicles in the adjacent lane was minimized); (2) over the lane-marking situation, in which one side of the vehicle was on the lane-marking and the vehicle didn’t encroach into the adjacent lane; and (3) transgressing-lane situation, in which any parts of the vehicle passed the lane marking (in such a situation, the vehicle has the greatest impact on the vehicles in the adjacent lane(s)). The three situations can be presented as the following equations:

(1) $$if\;|l_{d}|＜\frac{l_{w}-v_{w}-m_{w}}{2},\;normal\;situation\;$$

(2) $$f\;\frac{l_{w}-v_{w}-m_{w}}{2}\leq |l_{d}|\leq \frac{l_{w}-v_{w}+m_{w}}{2},\;over-lane-marking\;situation$$

(3) $$if\;|l_{d}|＞\frac{l_{w}-v_{w}+m_{w}}{2}\;,\;transgressing-lane\;situation\;$$

where *l_d_* is the lane deviation of vehicle, *l_w_* is the lane width, *v_w_* is the vehicle width (1.8 m), and *m_w_* is the marking line width (0.15 m), as requested by Urban Expressway Design Specification in China (CJJ129-2009).

#### 2.4.2. Subjective Data

After driving, each participant completed a subjective questionnaire about subject-based information such as age, gender, driving experience, perception and assessment of the simulation. Subjective perceptions in the questions were weighted with a score of 1 to 3, based on the impact level of the independent variable, such as no impact, 1, slight impact, 2, or significant impact, 3.

### 2.5. Experiment Design and Procedure

The experiment consisted of four parts: (1) general instructions; (2) a 15 min training session in the simulator; (3) driving in different scenarios, which are presented in Table 1; and (4) a questionnaire survey. The 15 min training session allowed participants to get familiar with the operation, and effectively eliminated the differences between participants with and without driving simulator experience.

Participants were also requested to maintain a reasonable and safe speed according to the road condition, and stay in the same lane throughout each scenario. Participants were asked to drive twice in each lane (left, middle and right lane) of each scenario. In order to alleviate simulator sickness, the driver would take a rest for three minutes after each scenario. Analysis of variance (ANOVA) with repeated measurements was used to analyze the effects of cross-section factors of the underground expressway on driving behavior.

To minimize the impact of the learning procedure in the simulator, the order of the test scenarios was randomized. In each scenario, the participant had 200 m to accelerate at the beginning, and 100 m to decelerate in the end. Data from these two sections were removed to exclude the starting and braking process.

## 3. Results and Analysis

### 3.1. Speed Results

The summary of the average travel speed and SD for driving on different lanes in each scenario is presented in Table 2.

#### 3.1.1. Effect of Lane Width on Speed

Results from ANOVA showed a significant effect of lane width on average speed (F = 1234.369, *p* < 0.01). As expected, the results indicated that the average speed increased with the lane width. Participants drove significantly faster on the road with the lane width of 3.75 m compared to others. The box-plot shows the speed distributions in scenarios with the five lane widths (in 0.75 m shoulder width). With the same shoulder width of 0.75 m, the average speed increased from 60.01 km/h in the narrowest lane (2.85 m) to 88.05 km/h in the widest lane (3.75 m). The statistic results of driving speed (minimum, median, maximum, first and third quartile) also elucidated that the driving speed was positively correlated to the lane width. The reason might be that due to the decrease of perceived risk in wide lanes, drivers would take aggressive behaviors such as speeding.

Interestingly, from the results, operational speeds were always over the limit. For instance, although the design speed of a 3 m wide lane road is often less than 40 km/h in the design specifications of many countries [33], the operational speeds were in most cases over 60 km/h. This is consistent with the findings in [34].

#### 3.1.2. Effect of Lane Position on Speed

Results for the effect of the lane position on the speed, as presented in Table 2, differed from lane width cases. For example, the ANOVA result for the shoulder width of 0.75 m, as presented in Table 2, showed a significant difference in the average speed among three lane positions with the lane width of 2.85 m (F = 211.67, *p* < 0.01), 3 m (F = 169.78, *p* < 0.01) and 3.25 m (F = 12.30, *p* < 0.01). However, differences among average speeds in left, middle and right lane were found to be not significant with the lane width of 3.5 m (F = 0.90, *p* = 0.41) or 3.75m (F = 2.53, *p* = 0.11).

From Table 2, in most cases, the average speed in the left lane was the highest, while the average speed in the middle lane was the lowest. The reason might be that drivers on the left lane and right lane tended to encroach on the road shoulder when the lanes were narrow (they perceived to be driving in lanes “with extended width”); however, owing to the confined space, drivers on the middle lane had no extra space to compensate the lane width, thereby the speed of the middle lane was lower than the other two lanes. If the lanes were with enough width, participants felt it easy and safe to drive at a high speed confidently in all the three lanes, which was the reason why the differences were not significant among three lanes with a large lane width.

#### 3.1.3. Effect of Shoulder Width on Speed

**Figure 4:**
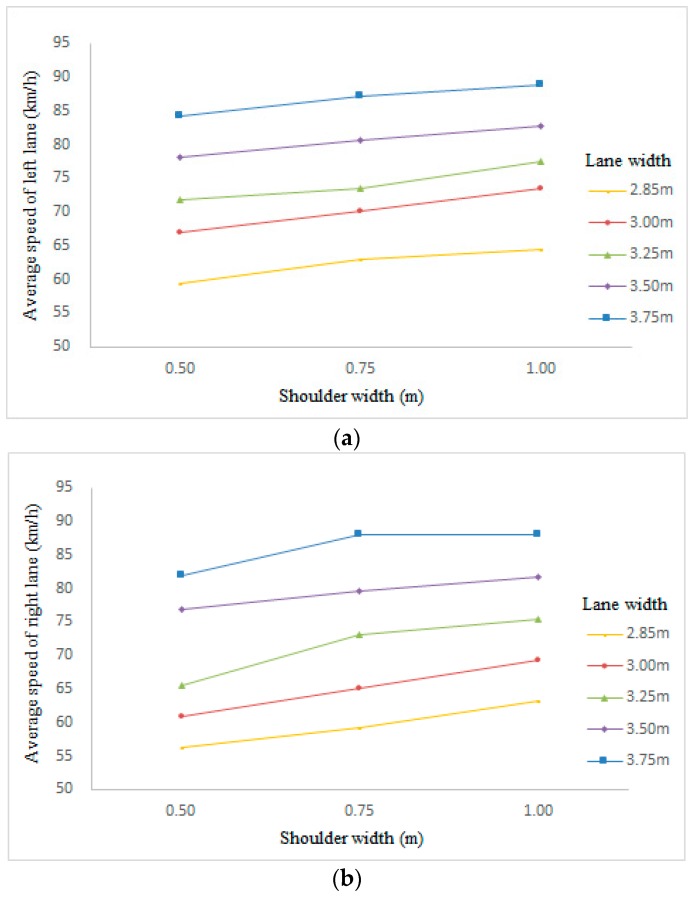
Effect of shoulder width on average speed in side lanes. (**a**) Effect of shoulder width on average speed in left lanes; (**b**) Effect of shoulder width on average speed in right lanes.

In general, shoulder width has a positive effect on speed. However, the significance of the effect varies from lanes. The effect of shoulder width on average speed of the middle lane was not significant (F = 1.17, *p* = 0.35), while it was significant on the speed of the left lane (F = 242.32, *p* < 0.01) and the right lane (F = 387.15, *p* < 0.01). Figure 4 shows the lines illustrating the trend of the average speed in the left lane and right lane with the increase of the shoulder width in different lane width cases. For both the left lane and the right lane, participants drove faster on the road with large shoulder width compared to that with a reduced one. Although the average speed increased with the lane and shoulder width, the speed variation (as presented in Table 2 and Figure 4) caused by changes in lane width was larger than that caused by changes in shoulder width. In other words, lane width had a greater effect on speeds than shoulder width.

### 3.2. Lane Deviation Results

Table 3 lists the summary of lane deviation for tests on different lanes.

#### 3.2.1. Effect of Lane Width on Lane Deviation

**Figure 5:**
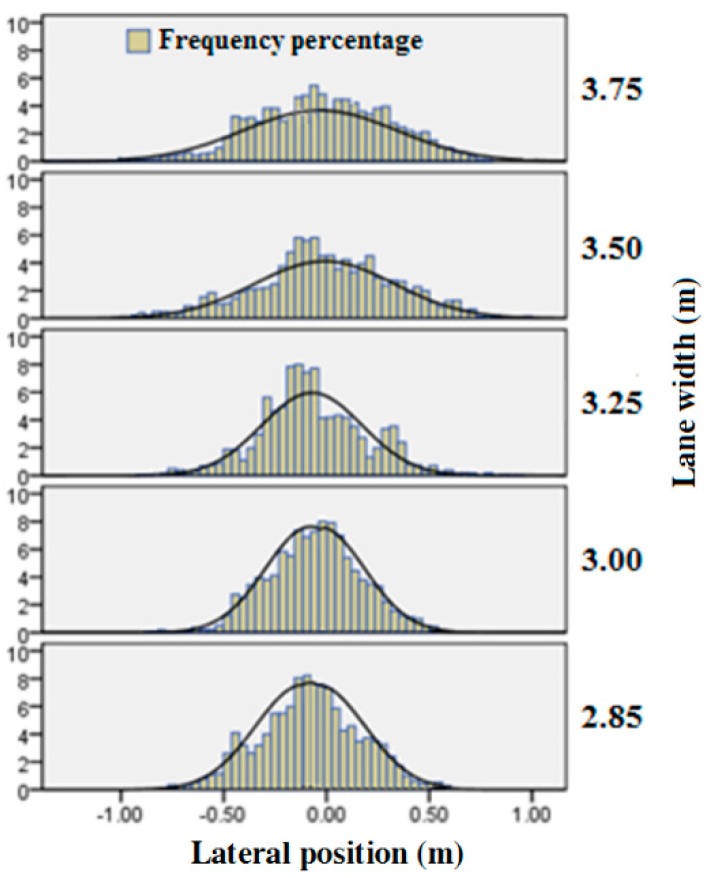
Effect of lane width on lane deviation.

Results of ANOVA showed a significant effect of lane width on lane deviation (F = 96.93, *p* < 0.01). According to the definition, the closer the lane deviation value was to zero, the nearer the vehicle track was to the lane centerline. If the absolute value of lane deviation was over half of the lane width, the vehicle had deviated into the other lanes. The frequency percentage of lane deviation obeyed normal distribution (see Figure 5). From Table 3 and Figure 5, one can see that the range of lane deviation increased with increment of lane width, which is in alignment with the fact that the vehicles have a wider space to manage with a wider lane width.

As mentioned, the participants were asked to keep driving on the test lane. According to the result (the extrema values), most of the participants could keep the lane position well and few vehicles deviated into the other lanes.

#### 3.2.2. Effect of Lane Position on Lane Deviation

The effect of lane position on lane deviation was found to be large and significant (F = 216.17, *p* < 0.01).

**Figure 6:**
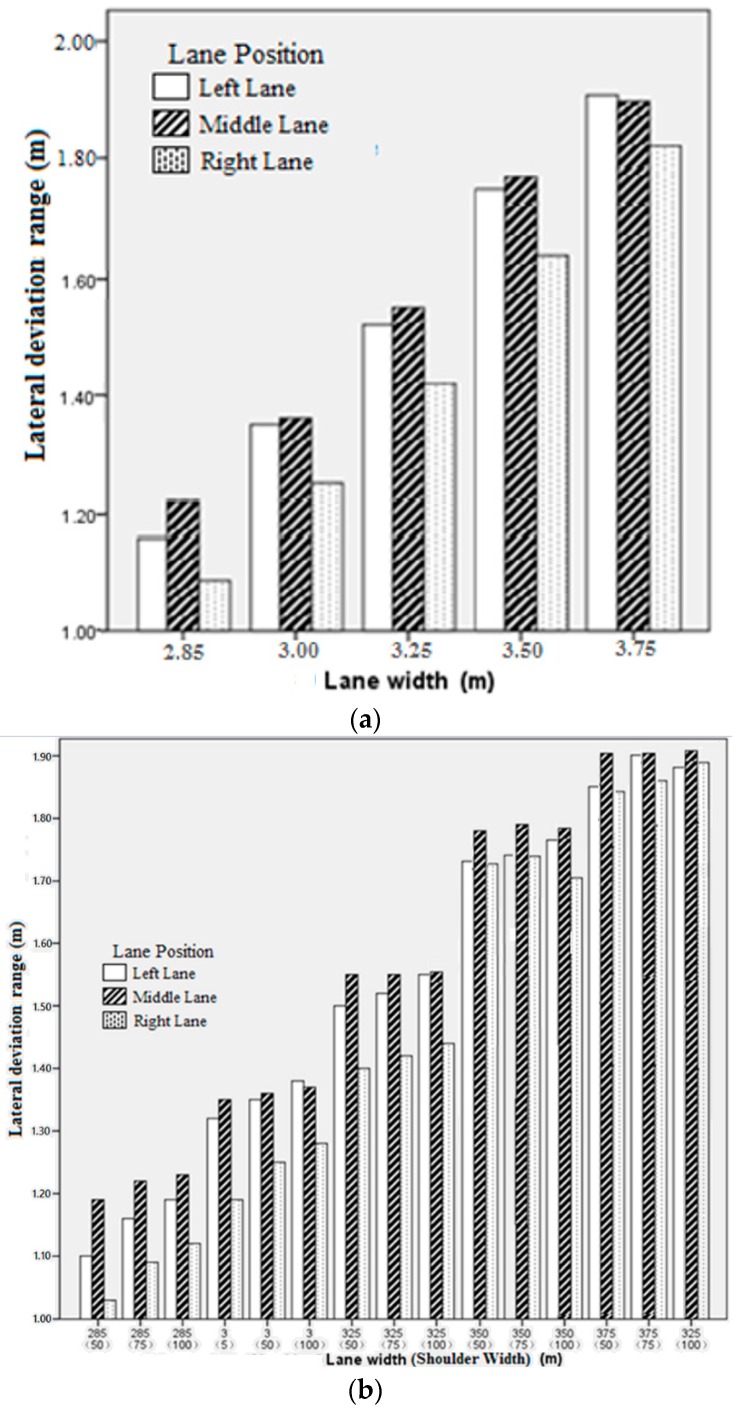
Effect of lane deviation range, for combination of lane width, shoulder width and lane position. (**a**) The effect of lane width on Lane deviation range; (**b**) Lane deviation range in different combination.

From Table 3, in general, the mean values of lane deviation of the left and middle lane were negative and were positive in the right lane cases. This indicated that drivers tended to drive far against the wall when driving on roads with small lane width. On roads with wider lanes, drivers could keep in the requested driving lane easily, which, especially, is more significant in the lane deviation values in left and right lanes, and could be illustrated by the trend of the change in the mean values of lane deviation listed in Table 3. With the increase of lane width, the mean lane deviation values in all lanes got close to zero. Furthermore, the lane deviation range in the right and left lanes are lower than that in middle lane (Figure 6a) proving the existence of side wall effect, which makes drivers keep far against the wall when driving on the side lane. From the results, the higher lane deviation range in the left lane over that in the right lane shows that the effect on the vehicles in the right lane was greater than that in the left lane.

#### 3.2.3. Effect of Shoulder Width on Lane Deviation

Results of ANOVA showed that shoulder width had no significant effect on lane deviation of the middle lane (F = 2.17, *p* = 0.12), while its effect on lane deviation of the left lane (F = 66.17, *p* < 0.01) and right lane (F = 45.31, *p* < 0.01) was significant. Lane deviation range was calculated based on the equation: Range = Maximum−Minumum. The results from Figure 6b clearly show an increment in lane deviation range in the left and right lanes with the increase in shoulder width.

The effects of lane width and lane position on lane deviation were also further verified; and the variation of lane deviation range caused by changes in lane width was larger than that caused by changes in shoulder width.

### 3.3. Subjective and Objective Evaluation

#### 3.3.1. Subjective Evaluation

The subject evaluation was conducted based on the perception of the participants, the study of which could help investigate the gap between perception and the real traffic environment. From questionnaire survey results, lane width had a great effect on driving performance, with an average impact score of 2.85 (out of 3). Three quarters of the 24 participants found that shoulder width affects their decisions to maintain the speed and the lateral position (the average score was 2.25). The results on the effect of lane width and shoulder width show a high alignment of the participant perception with the real road simulation data. However, the results surprisingly showed that only less than 20% of the participants felt that the lane position had a great effect on their driving maneuver, and the average score was 1.3. This is in contrast to the analysis from the objective data where their speed and lane deviation were found to be significant affected by lane position. From the comparison between subjective evaluation and actual data, driving behavior did not always match their perception of the road. 

#### 3.3.2. Objective Evaluation

**Figure 7:**
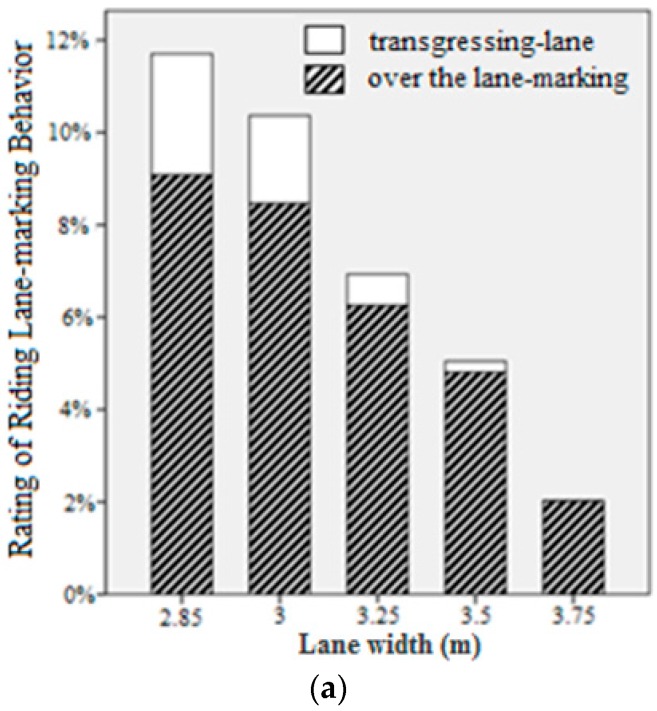

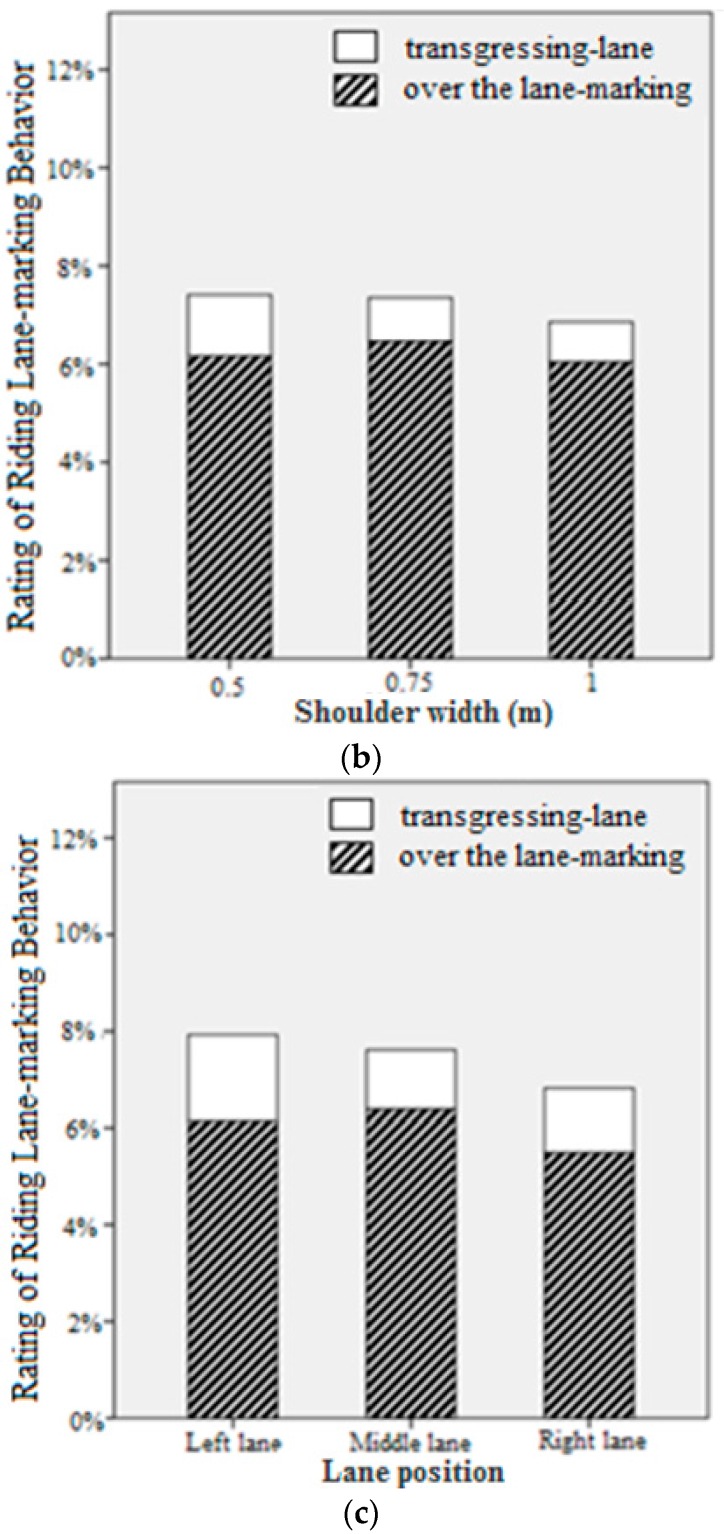
Effects on objective safety, for lane width, shoulder width and lane position. (**a**) Effect of lane width on objective; (**b**) Effect of shoulder width on objective; (**c**) Effect of lane position on objective.

Figure 7 presents results for the rating of riding lane marking behavior in different test cases. As expected, objective safety was the highest with the lane width of 3.75 m and shoulder width of 1 m (Figure 7a,b) except in the case of constantly weaving. Meanwhile, vehicles in the middle lane had the highest rate of over the lane marking in most of the cases; however, they had the lowest rate of transgressing-lane. The reason is that drivers on the middle lane were less affected by side wall. They would have the highest deviation range. At the same time, they could stay in the requested driving lane well and seldom occupied other lanes. Vehicles on the left lane had the highest rating of transgressing-lane as shown in Figure 7c indicating that they tended to drive on the road shoulder.

## 4. Discussion

Contrary to past studies, which focus on only one factor, this study used a counterbalanced experimental design in order to consider all the key factors. It included 15 possible combinations of the three independent measures. In general, all three factors including lane width, shoulder width and lane position significantly affect driving behaviors in terms of speed and lane deviation. From the results, in the underground urban expressway, wider lane and shoulder width gave the drivers more freedom in lateral space, higher perceived safety and objective safety, and increased the driving speed. Practically, operating speeds were found to be always over the speed limit, leading to an increase in accident risks. These findings are generally consistent with past studies focusing on regular urban expressways, indicating that the advanced simulator potentially provides an effective method in investigating the impact of road design specifications on driver behavior, especially in a more comprehensive way with consideration of multiple factors.

Meanwhile, by investigating the impact of multiple factors using the counterbalanced experimental design, specific results from different combination cases were extracted and some interesting findings that have not been investigated in past studies can be drawn:

- Shoulder width did not have significant impact on average speed of vehicles in the middle lane. For the left and right lane, the effects of shoulder width on driving behavior were partially similar with lane width.
- With a narrow road shoulder, drivers on the right or left lane tended to drive far away from the wall, with some deviating into the other lane. As the shoulder got wider, the impact became insignificant.
- Participants drove faster and had a larger lane deviation in a wide shoulder compared to in a small one.
- Driver behavior was more affected by lane width compared to shoulder width, which is in line with driver perception as suggested in [25], where drivers determined the lateral position mainly by recognizing the position of the marking.
- Wide shoulder would bring the drivers high perception of safety and reduce the proportion of dangerous displacement, e.g., transgressing-lane driving.

These findings indicate that the approach of using the advanced driving simulator provides a more detailed and potentially more precise way for validating the factors of underground expressway designs and related specifications. Several recommendations for design specifications can be drawn from the study in order to strengthen safety in underground urban expressways. For example, setting a reduced speed limit of the left lane could be used to slow down the vehicles speed and reduce the lane deviation on the left lane. It also provides evidence for setting specific limit speed values for different lanes. Additionally, a suitable roadside landscape and rumble strips could help reduce the speed variability between the middle lane and other two lanes. Moreover, setting a proper shoulder width could help reduce the lane deviations, especially on the left and right lanes.

However, results from the objective measure and the subjective perception show some inconsistency between driver perception and real road situation. Lane position was tested to have a significant impact on driving behavior but only 20% realized that they were affected by their lane position. Reasons for this might be that: (1) the different impact of lane position is generated mainly from other factors, such as the extended maneuvering space with the existence shoulder, shoulder width and lane width, which is more noteworthy to drivers; (2) though greatly reduced by using the advanced simulation, difference of driver perception in virtual road environment and real road context still exists.

## 5. Conclusions

This study investigated the effect of three independent road alignment variables—lane width, lane position and shoulder width—on driving behavior in urban bypass expressway using the advanced driving simulator. Lane width, lane position and shoulder width were found to have a significant impact on driving behavior in terms of speed and lane deviation. A high-end simulator was used in the study to build a driving environment that is very close to a real-world environment. Results were found to be reasonable, indicating that using the advanced driving simulator could be a practical way to validate the design and safety of an underground expressway in light of different affecting factors.

As the main contributions, the study introduced the use of the advanced driving simulator in investigating the impact of the key cross-section factors of an underground urban expressway, which has been less studied in the past. This method provides a potentially more precise and detailed way to explore driver behavior and road safety on underground urban expressways more comprehensively. Additionally, the method of using the advanced driving simulation can be practically used to validate and amend the current urban underground road design specifications and standards, such as the design of lane width, shoulder width and lane marking and the set of speed limit.

Using the driving simulator could be a very practical way to assess design and safety before spending heavily in construction, since it provides the possibility of detecting unreasonable design aspects and potential safety issues at the beginning; however, the gap between the simulated environment and the real-world environment cannot be ignored and warrants further investigation. The limitations of a driving simulator, such as simulator sickness, ecological validity in laboratory-based study, driver motivation, and level of perceived risk in a simulated environment, should also be synthetically considered according to the research object in order to minimize their influence. The present work aims to study how drivers’ behavior is influenced by cross-sections of an underground expressway. To avoid the impact of other factors on this research object, the experimental scenarios were defined as no lane changing and no other traffic in the same lane. Drivers’ behavior, such as car following and lane changing, should be studied in the future. In addition, to comprehensively explain the driving behavior in underground urban expressway, research on drivers’ psychology and physiology, such as workload, eye-movement, heart rate and blood pressure, would also be interesting topics in driving behavior and road safety studies. Because the entire experimental session was excessively long and monotonous, it could impact negatively on driver behavior, motivation and psychological state. The experimental approach will be further studied and optimized in the future.

## Figures and Tables

**Table 1:** Parameters for each scenario.

No. of Scenario	1	2	3	4	5	6	7	8	9	10	11	12	13	14	15
Lane position	l	m	r	l	m	r	l	m	r	l	m	r	l	m	r
Lane width (m)	2.85			3.00			3.25			3.50			3.75		
Shoulder width (m)	0.5	0.75	1	0.5	0.75	1	0.5	0.75	1	0.5	0.75	1	0.5	0.75	1

Note: for lane position, l indicates left lane, m indicates middle lane and r is right lane.

**Table 2:** Mean value and standard deviation (SD) of speed, for different scenarios.

Lane Width (m)	Shoulder Width (m)	Average Speed (km/h)	Mean Value in Different Lanes (km/h)					
			Left Lane	SD	Middle Lane	SD	Right Lane	SD
2.85	0.5	57.23	59.29	7.43	56.12	7.11	56.18	6.45
	0.75	60.01	62.98	8.21	56.51	9.86	59.29	10.31
	1	61.47	64.36	9.87	57.04	10.67	63.19	9.79
3	0.5	63.82	67.01	9.36	64.23	9.41	60.78	8.18
	0.75	67.51	70.19	8.96	64.72	7.55	65.14	11.27
	1	69.29	73.58	9.84	65.47	8.64	69.38	8.17
3.25	0.5	69.06	71.79	9.57	70.1	10.39	65.46	9.96
	0.75	73.17	73.53	8.51	71.52	8.51	73.06	8.55
	1	74.83	77.51	10.46	71.88	10.33	75.32	11.11
3.5	0.5	77.58	78.14	7.65	79.45	9.75	76.85	10.73
	0.75	80.11	80.61	11.88	79.45	10.37	79.69	8.15
	1	81.56	82.76	9.31	79.84	8.86	81.77	8.97
3.75	0.5	84.13	84.16	12.86	86.15	8.25	81.92	11.65
	0.75	88.05	87.23	13.37	88.93	11.01	87.98	14.42
	1	88.10	88.91	9.75	87.56	10.16	88.03	10.74

**Table 3:** Mean, standard deviation and extrema of lane deviation (0.75 m shoulder).

Lane Width (m)	Lane Position	Mean Value (m)	SD	Minimum (m)	Maximum (m)
2.85	Left	−0.16	0.32	−0.70	0.46
	Middle	−0.05	0.34	−0.63	0.59
	Right	0.12	0.32	−0.44	0.65
3	Left	−0.13	0.35	−0.76	0.59
	Middle	−0.03	0.36	−0.7	0.66
	Right	0.1	0.33	−0.55	0.70
3.25	Left	−0.11	0.38	−0.85	0.67
	Middle	−0.03	0.41	−0.8	0.75
	Right	0.08	0.35	−0.62	0.80
3.5	Left	−0.08	0.42	−0.95	0.80
	Middle	−0.01	0.47	−0.9	0.87
	Right	0.07	0.40	−0.76	0.88
3.75	Left	−0.06	0.45	−1.01	0.9
	Middle	−0.02	0.49	−0.95	0.95
	Right	0.05	0.42	−0.85	0.97
